# Label free, capillary-scale blood flow mapping in vivo reveals that low-intensity focused ultrasound evokes persistent dilation in cortical microvasculature

**DOI:** 10.1038/s42003-024-07356-2

**Published:** 2025-01-06

**Authors:** YuBing Y. Shen, Jyoti V. Jethe, Ashlan P. Reid, Jacob Hehir, Marcello Magri Amaral, Chao Ren, Senyue Hao, Chao Zhou, Jonathan A. N. Fisher

**Affiliations:** 1 Department of Physiology, New York Medical College, Valhalla, NY USA; 2 Department of Biomedical Engineering, Washington University in St Louis, St. Louis, MO USA; 3 Biomedical Engineering, Universidade Brasil, San Paulo, SP Brazil; 4 Imaging Science Ph.D. Program, Washington University in St Louis, St. Louis, MO USA; 5 Department of Electrical & Systems Engineering, Washington University in St Louis, St. Louis, MO USA

**Keywords:** Neuro-vascular interactions, Optical imaging

## Abstract

Non-invasive, low intensity focused ultrasound is an emerging neuromodulation technique that offers the potential for precision, personalized therapy. An increasing body of research has identified mechanosensitive ion channels that can be modulated by FUS and support acute electrical activity in neurons. However, neuromodulatory effects that persist from hours to days have also been reported. The brain’s ability to provide blood flow to electrically active regions involves a multitude of non-neuronal cell types and signaling pathways in the cerebral vasculature; an open question is whether persistent effects can be attributed, at least partly, to vascular mechanisms. Using an in vivo optical approach, we found that microvasculature, and not larger vessels, exhibit significant persistent dilation following sonication without the use of microbubbles. This finding reveals a heretofore unseen aspect of the effects of FUS in vivo and indicates that concurrent changes in neurovascular function may partially underly persistent neuromodulatory effects.

## Abstract (web summary)

Capillary-scale blood flow mapping in vivo reveals that low intensity focused ultrasound evokes persistent dilation in cortical microvasculature

## Introduction

Low-intensity focused ultrasound (FUS) is an emerging technology for non-invasive neuromodulation. Compared with other non-invasive modalities such as transcranial magnetic stimulation (TMS) or transcranial direct current stimulation (tDCS), FUS offers the potential for restricting modulatory stimuli to within volumes that are ~3–5 orders of magnitude smaller and with millimeter-scale cross-sectional focal area^1–5^. These advantages in resolution could minimize off-target effects and enable more precise and personalized treatment approaches. In human subjects, FUS has been explored as a therapeutic for a wide range of conditions such as stroke^6^, Alzheimer’s disease^7,8^, essential tremor^9,10^, Parkinson’s disease^11,12^, as well as chronic pain^13^. Beyond therapy, FUS has been shown to acutely modify sensory-evoked neural responses in small animal models^14^, swine^15^, nonhuman primates^16^, and humans^17^. Given that FUS has the capability of evoking sensations including somatosensory^18–22^ and visual^23,24^, this modality has presented new stimulus methods for brain-computer-interface applications^25–27^.

Central to wider clinical adoption is a clearer understanding of dosage and effects. The design of stimuli and dosing parameters is quite broad, and changing specific parameters, such as duty cycle, has been shown to qualitatively alter the brain’s response and the duration of these effects^28^. For example, modifying the duty cycle can switch the effects from excitatory to inhibitory, even if the average pressure remains constant^29^. Recent work on mechanisms has provided compelling evidence that FUS elicits action potentials in neurons by activating mechanosensitive Ca^2+^ channels that include TRPP1, TRPP2, TRPC1, and Piezo1^30^. FUS stimulation has also been shown to increase Ca^2+^ influx in astrocytes by opening TRPA1 channels, resulting in glutamate release through Bestrophin-1 (Best1) channels^31^.

Beyond acute electrophysiological effects, FUS can elicit persistent plasticity^2,16,32^ up to weeks, in some studies^33,34^. In terms of underlying mechanisms for these chronic effects, FUS appears to modulate the function of NMDA receptors^35^, a key contributor to many forms of synaptic plasticity^36–38^. Zhao et al.^8,39^ proposed that increases in brain-derived neurotrophic factor, glial cell line-derived neurotrophic factor, and vascular endothelial growth factor induced by FUS might explain the observed acceleration in dendritic spine growth over a 28-day treatment period.

Sonication-evoked cerebrovascular effects and hemodynamics, generally considered secondary to neuronal activity^40^, could also underly at least some of the observed neuromodulatory effects. Neuronal activity triggers changes in cerebral blood flow and volume that are localized to the same regions that exhibit electrical activity. This phenomenon, termed neurovascular coupling, is the result of an intricate choreography of multiple cell types and signaling mechanisms^41^ and perturbing vasculature would impact neuronal function. In fact, pathological perturbation of even single capillaries can cause larger-scale changes in blood flow^42^ and may result in microvascular ischemia^43^. In terms of potential mechanisms, some of the same mechanosensitive channels found to be critical for FUS-induced neural effects are also expressed in vasculature. TRPC1, for example, is expressed in vascular endothelial cells^44–46^, TRPP1 in vascular smooth muscle cells^47–49^, and Piezo1 is expressed in both cell types and also plays a critical role in vascular development and remodeling^50–52^. Because hemodynamic responses lag neural activation, vascular effects could potentially contribute to persistent apparent neuroplasticity.

The impact of FUS on cerebral hemodynamics has been studied using invasive and non-invasive methods. Functional magnetic resonance imaging (fMRI), for example, has been instrumental in noninvasively exploring the deep brain effects of FUS neuromodulation as well as its viability in larger brains including humans. fMRI has revealed FUS-induced hyperaemia both cortically and subcortically by means of volumetric blood-oxygen-level-dependent (BOLD) signals^16,22^. Near-infrared spectroscopy (NIRS)^53^, another non-invasive approach for monitoring cerebral hemodynamics, has been used to measure hemodynamic signals evoked by administering FUS to the somatosensory cortex^54^. While generally lacking the spatial resolution of fMRI, NIRS can monitor hemodynamics with much higher temporal resolution^55^. Kim et al. found that the FUS-induced hemodynamic response, measured in terms of inferred changes in oxy- and deoxyhemoglobin concentrations, largely resolves within 20 seconds. That timescale is similar to sensory-evoked hyperaemia measured using NIRS^54^. In contrast to non-invasive methods that have a lower spatial resolution, invasive techniques such as laser speckle contrast imaging and optical intrinsic signal imaging (OISI Yuan et al., 2023;) can detect FUS-induced changes in blood flow and dilation in specific vessels^56,57^ and optical intrinsic signal imaging (OISI Yuan et al., 2023) have resolved FUS-induced flow changes and dilation in individual vessels. Mirroring the NIRS findings, these studies have similarly observed FUS-induced hemodynamic responses in individual vessels largely subside within ~10 seconds.

While the results of FUS-associated hemodynamic measurements seemingly suggest that acute effects are likely of neuronal or astrocytic origin and that persistent neuromodulatory effects do not manifest in prolonged vasomodulation, none of these experiments has heretofore directly resolved the impact of FUS alone on microvasculature. For widefield optical techniques such as laser speckle and OISI, this is partly owing to relatively poor axial resolution due to the use of low-numerical aperture lenses. Measuring the functional response in small vessels is critical because microvascular branches ~3−15 μm in diameter, which include capillaries, small arterioles, and venules, are at the frontline for neurovascular coupling^41,58^ and also likely easier to perturb mechanically by FUS^59^.

To obtain insight into a potential microvascular reflection of persistent effects following sonication, we developed a label-free optical approach for interrogating dilation in vasculature down to capillary scale in vivo directly at the site of ultrasound delivery. Our apparatus used a custom spectral domain optical coherence tomography angiography (OCT-A) device that resolves vasculature based on light scattering changes due to moving red blood cells. Compared with other optical modalities for resolving blood flow at microvascular scales, OCT-A offers the advantage of being label-free, in contrast with other optical techniques, such as two-photon microscopy, which requires intravenous tracers or targeted expression of fluorescent reporters. Additionally, because the depth resolution in OCT is limited by the characteristics of the broadband light source rather than an objective’s optical depth of field^60^, relatively low-numerical aperture, long working distance lenses can be used to survey large brain regions without sacrificing depth resolution. Integrating this with a wide-aperture ring transducer whose acoustic and optical foci were co-localized, we were able to obtain a direct glance into the effects of FUS on cortical microvasculature.

## Results

### In situ, label-free microvascular-level measurements of dilation during FUS neuromodulation

**Fig. 1:**
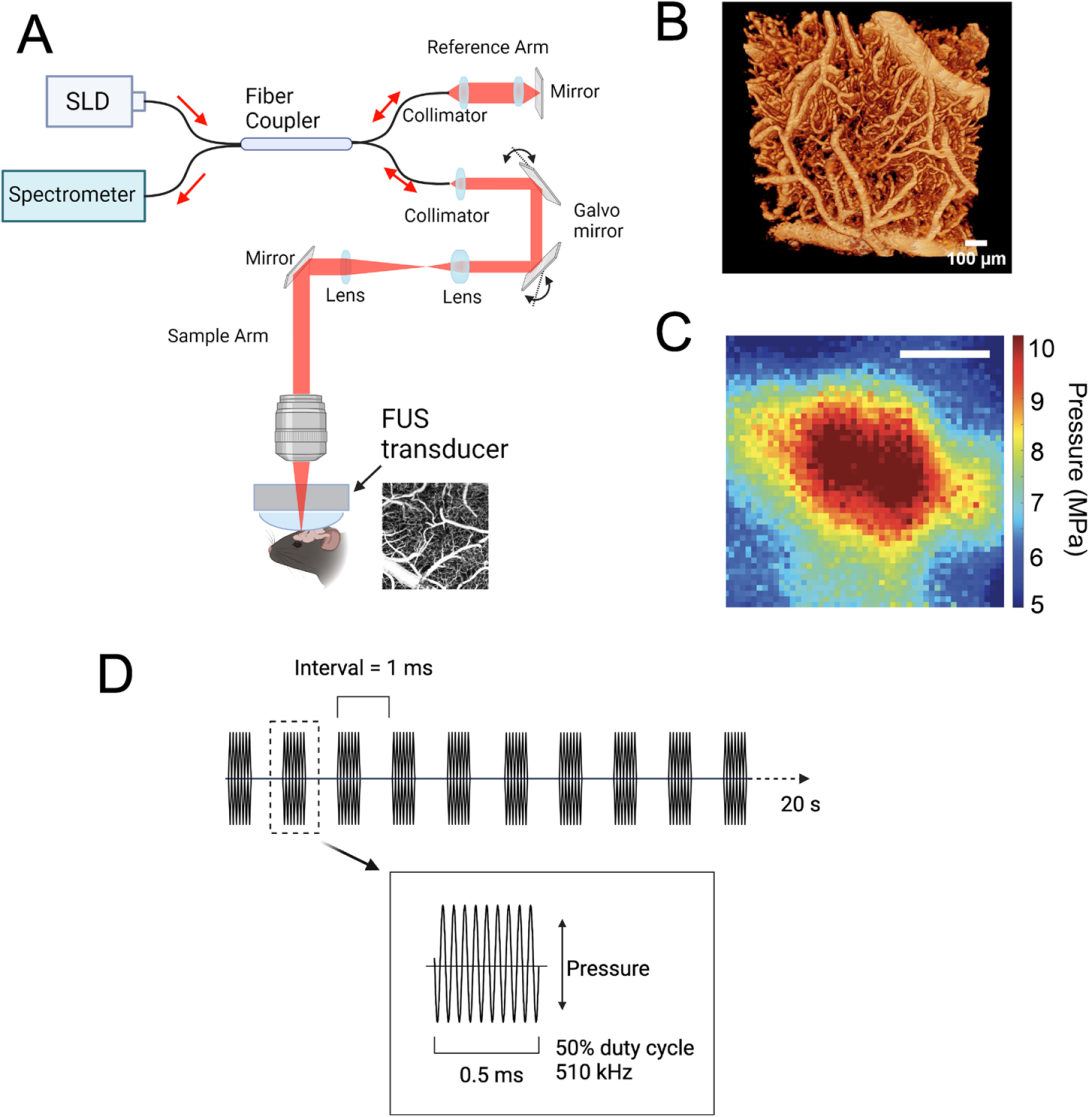
Combined optical coherence tomography angiography (OCT-A) and FUS for observing multi-scale vascular responses to sonication. **A** depicts a schematic for a custom frequency domain OCT-A apparatus with an in-line FUS ring transducer. The inset shows a sample 1 mm × 1 mm en face z-projection revealing flow patterns measured in somatosensory cortex. **B** shows a sample 3D surface mesh rendering of vascular architecture, as reconstructed from OCT-A imagery. **C** shows the lateral profile of the FUS beam (scale bar = 1 mm), and **D** illustrates the pulsed sonication protocol; sonication was delivered as a 20 s period of 510 kHz ultrasound pulsed at 1 kHz with 50% duty cycle.

Using a custom spectral domain OCT-A apparatus (Fig. 1A) that was able to compensate for long working distances that traversed ~10 cm of water within the ring transducer, we were able to obtain 3D portraits of the vasculature (Fig. 1B) with 2.48 × 3.48 μm (axial × lateral) resolution at depths upwards of 650 μm, label-free in wild-type mice. Whereas techniques such as MR-acoustic radiation force imaging can be used to localize the FUS focus in fMRI experiments^61^, a challenge for targeting FUS in epi-illumination optical systems in vivo is that the FUS beam is not intrinsically optically detectable. Because small positioning errors can lead to uneven sonication patterns within an optical field of view, we used a phased array ring transducer with a beam width larger than the analyzed zones (Fig. 1C) to ensure that our analysis was restricted to vessels that received the same FUS dosing. Additionally, as depicted in Supplementary fig. 2, the axial profile of the beam reveals an extruded focus whose intensity profile had a full-width-half-max of ~5 mm. The sonication focal profile would be minimally altered by tissue and, therefore, can be considered relatively uniform throughout the imaging depth.

**Fig. 2:**
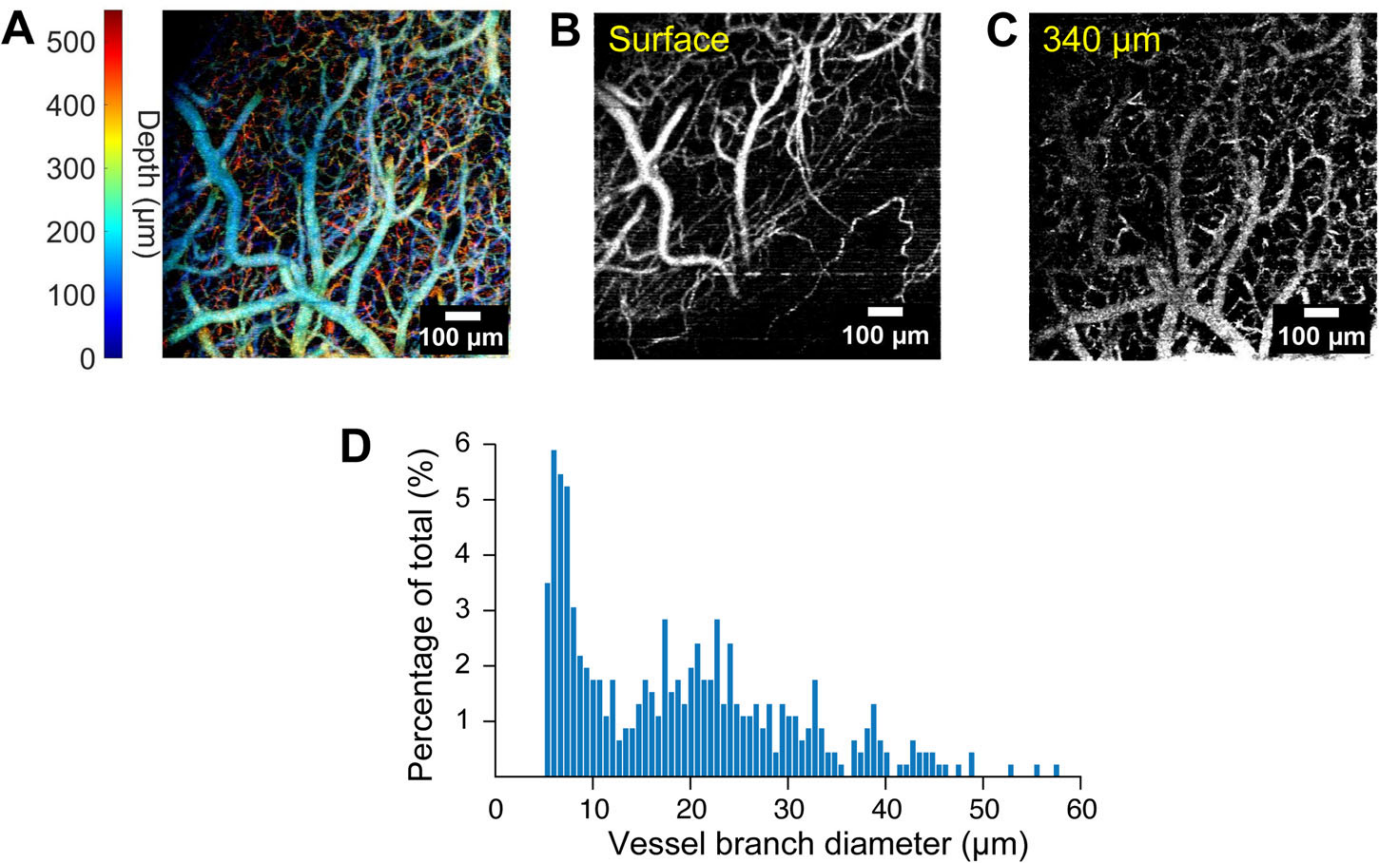
Depth-resolved flow patterns in the mouse somatosensory cortex. **A** shows data from all depths, which are here encoded by color. As is apparent, the deepest features resolve a fine mesh of narrow vessel branches. **B** and **C** show z-projections from narrow ranges of lateral slices centered at the cortical surface and ~340 μm below. The depth range for projections in **B** and **C** is ± ~2 μm axially. **D** depicts a histogram of vessel branch diameters derived from en face analysis. The histogram presents the summed results of 10 animals prior to any exposure to FUS.

Compared with vascular analysis and tabulation based on two-photon imaging^62^, the vessel morphology is of lower signal-to-noise. We, therefore, developed an image processing pipeline that was able to selectively segment vessel branches based on diameter, thereby permitting us to selectively analyze dilation dynamics in diverse vessel populations. Figure 2A presents a color-coded *z*-projection showing the full depth span of angiography data. Figure 2B, C shows *z*-projections taken over narrow ranges centered at the cortical surface and ~340 μm below, and Fig. 2D shows a histogram of vessel branch diameters derived from en face analysis. A visualization of the vessel segmentation process is depicted in Supplementary fig. 1.

### FUS elicits vascular dilation most prominently in small-diameter vessel branches

**Fig. 3:**
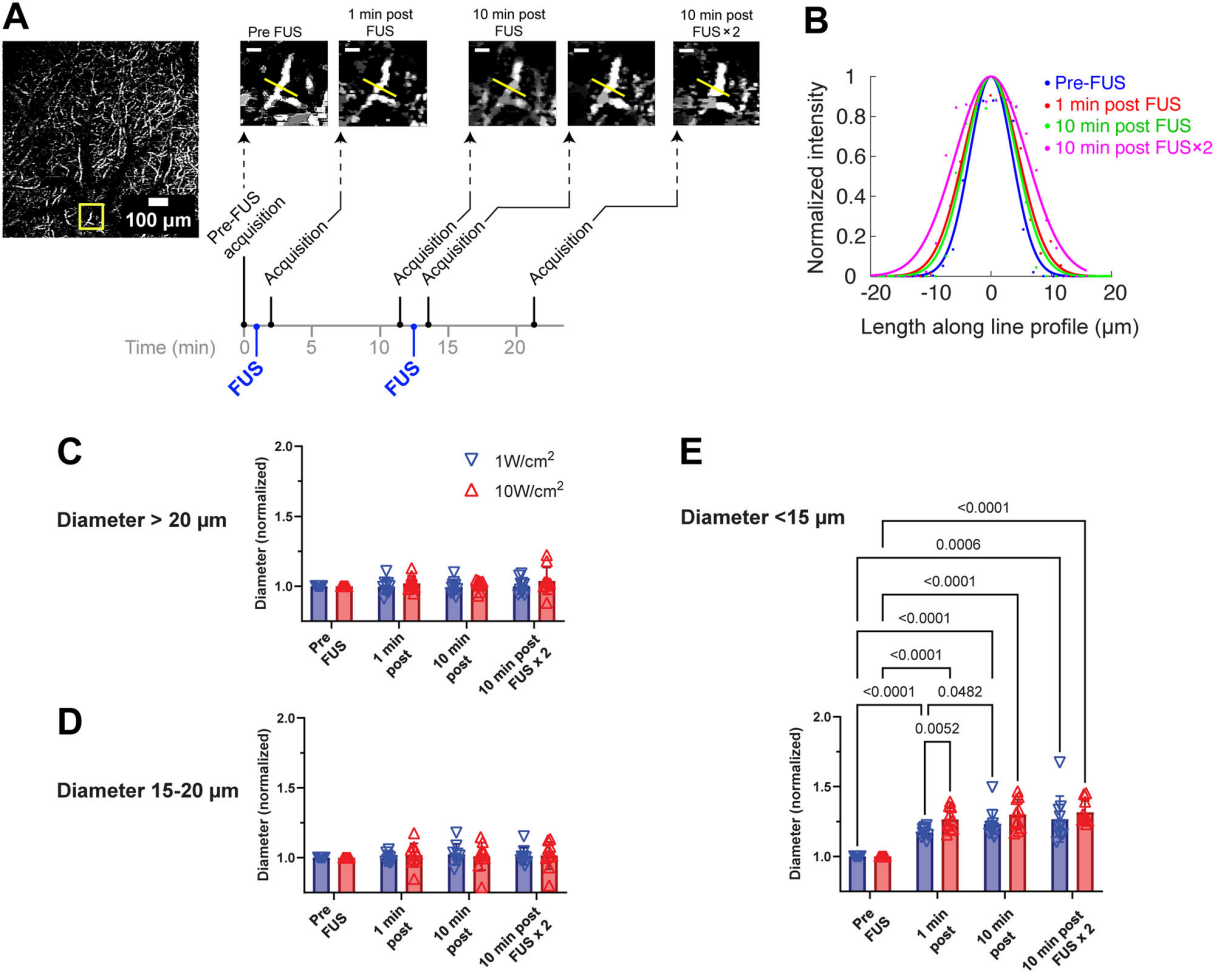
Dissecting vascular effects of FUS in vivo. **A** illustrates the sonication and imaging protocol in the form of a timeline and shows OCT-A monitoring in a single vessel branch during the course of consecutive doses of FUS. The yellow square in the left-most image shows a region that is expanded in images to the right. The dimensions for the full image on the left are 1 mm × 1 mm, and the dimensions image for smaller images on the right are 110 μm × 110 μm (scale bar: 20 μm). Sonication consisted of pulse bursts that were delivered at a rate of 1 kHz over a period of 20 sec. **B** shows Gaussian fits to the intensity profiles measured across the yellow lines shown in the smaller image panels of **A**; the dots show actual intensity values, and are derived from the one representative animal whose images are shown in **A**. The intensity profiles were normalized to visualize changes in diameter, which can be approximated by the full-width-half max of the Gaussian fits. **C**–**E** depict normalized changes in vessel branch diameters, averaged over all animals (*N* = 10 exposed to 1 W/cm^2^, *N* = 10 exposed to 10 W/cm^2^), and are analyzed in separate groups based on pre-FUS diameter ranges: >20 μm (**C**), 15–20 μm (**D**), and <15 μm (**E**). Vessel branches of pre-FUS diameter <15 μm, but not larger vessels, displayed significant increases in diameter that persisted beyond 10 minutes. Values displayed atop brackets in **E** report *P* values that were below 0.05, obtained using a two-way ANOVA and Fisher’s Least Significant Difference test. Individual data points correspond to results from individual animals.

Averaging data from the sonicated animals, excluding those used solely for immunohistochemical analysis, sonication at 510 kHz FUS (50% duty cycle, 1 kHz pulse repetition) applied at spatial-peak pulse-average intensities (*I*_sppa_) of 1 W/cm² (*N* = 10) and 10 W/cm² (*N* = 10) for 20 seconds (Fig. 3A, B) resulted in small, non-significant changes in the diameter of vessel branches greater than 15 μm, but caused significant dilation in smaller vessel branches (Fig. 3C–E and Table 1). Prior to FUS, the average diameter of the small vessel population was 6.2 ± 1.3 μm (mean ± standard deviation); within 1 minute following 1 W/cm^2^ sonication, this vessel population exhibited a 17% ± 3% increase in diameter (*P* < 0.0001, using a two-tailed paired Student’s *t* test). Sonicating at a higher intensity (10 W/cm^2^) yielded a greater dilation of 27% ± 8% (*P* < 0.0001), which was significantly different from the result of 1 W/cm^2^ (*P* = 0.0052 via two-way ANOVA and Fisher’s Least Significant Difference test). Following an initial sonication of 1 W/cm^2^, but not 10 W/cm^2^, vessel branches <15 μm exhibited a statistically significant further increase of 4% 10 minutes later (*P* = 0.048). Paired t-test statistics are presented in Table 1, while two-way ANOVA statistics are displayed in Fig. 3C–E.

**Table 1:** Analysis results from FUS treatment averaged among all experiments

Diam. range	FUS intensity (*I*_sppa_)	Diameter pre-FUS × 1 (μm)	Normalized diameter, 1 min post FUS × 1	Normalized diameter, 10 min post-FUS	Normalized diameter, 10 min post FUS × 2	*P* value of post FUS × 1 comparison with pre-FUS
All vessels	Sham (*n* = 3)	18.70 ± 19.79	1.00 ± 1.01	1.08 ± 1.05	1.04 ± 1.08	-
All vessels	1 W/cm^2^ (*n* = 10)	16.27 ± 16.01	1.05 ± 0.92	1.06 ± 0.91	1.06 ± 0.93	0.28
All vessels	10 W/cm^2^ (*n* = 10)	19.40 ± 14.39	1.02 ± 0.73	1.01 ± 0.71	1.01 ± 0.69	0.77
>20 μm	Sham	38.94 ± 23.27	0.98 ± 0.58	0.96 ± 0.58	0.98 ± 0.60	-
>20 μm	1 W/cm^2^	35.25 ± 19.16	1.00 ± 0.05	0.99 ± 0.05	1.00 ± 0.05	0.64
>20 μm	10 W/cm^2^	31.63 ± 13.43	1.02 ± 0.05	1.01 ± 0.04	1.04 ± 0.09	0.87
15–20 μm	Sham	17.28 ± 1.35	1.01 ± 0.08	0.97 ± 0.06	0.99 ± 0.06	-
15–20 μm	1 W/cm^2^	17.66 ± 1.38	1.00 ± 0.03	1.02 ± 0.07	1.01 ± 0.06	0.6
15–20 μm	10 W/cm^2^	17.57 ± 1.40	1.02 ± 0.08	1.01 ± 0.09	1.02 ± 0.09	0.45
<15 μm	Sham	6.50 ± 1.58	0.99 ± 0.01	1.00 ± 0.01	1.00 ± 0.004	-
<15 μm	1 W/cm^2^	6.20 ± 1.27	1.17 ± 0.03	1.23 ± 0.10	1.27 ± 0.16	**8.6E-26**
<15 μm	10 W/cm^2^	7.48 ± 2.24	1.27 ± 0.08	1.30 ± 0.1	1.32 ± 0.08	**3.6E-08**

Values following treatment are reported as normalized diameters. The FUS × 1 and FUS × 2 refer to the first and second sonication doses (see timeline in Fig. 3D). In addition to the changes summed over all vessel branch diameters, analysis is reported for selected diameter ranges. *P* values were obtained using a paired, two-tailed Student’s *t* test that compared individual animals. *P* values below 0.05 are emphasized with bold text. Diameter values are reported as mean ± standard deviation.

Vessel segmentation and analysis at the microvascular-level also enabled us to catalog and track individual vessel branches; Fig. 3D, E follow the dilation of one tracked sample vessel branch following sonication. In sham experiments (*N* = 3), vessel branches, regardless of diameter range, did not exhibit significant changes at any time point (Table 1).

### Depth-dependent dilation effects of FUS

**Fig. 4:**
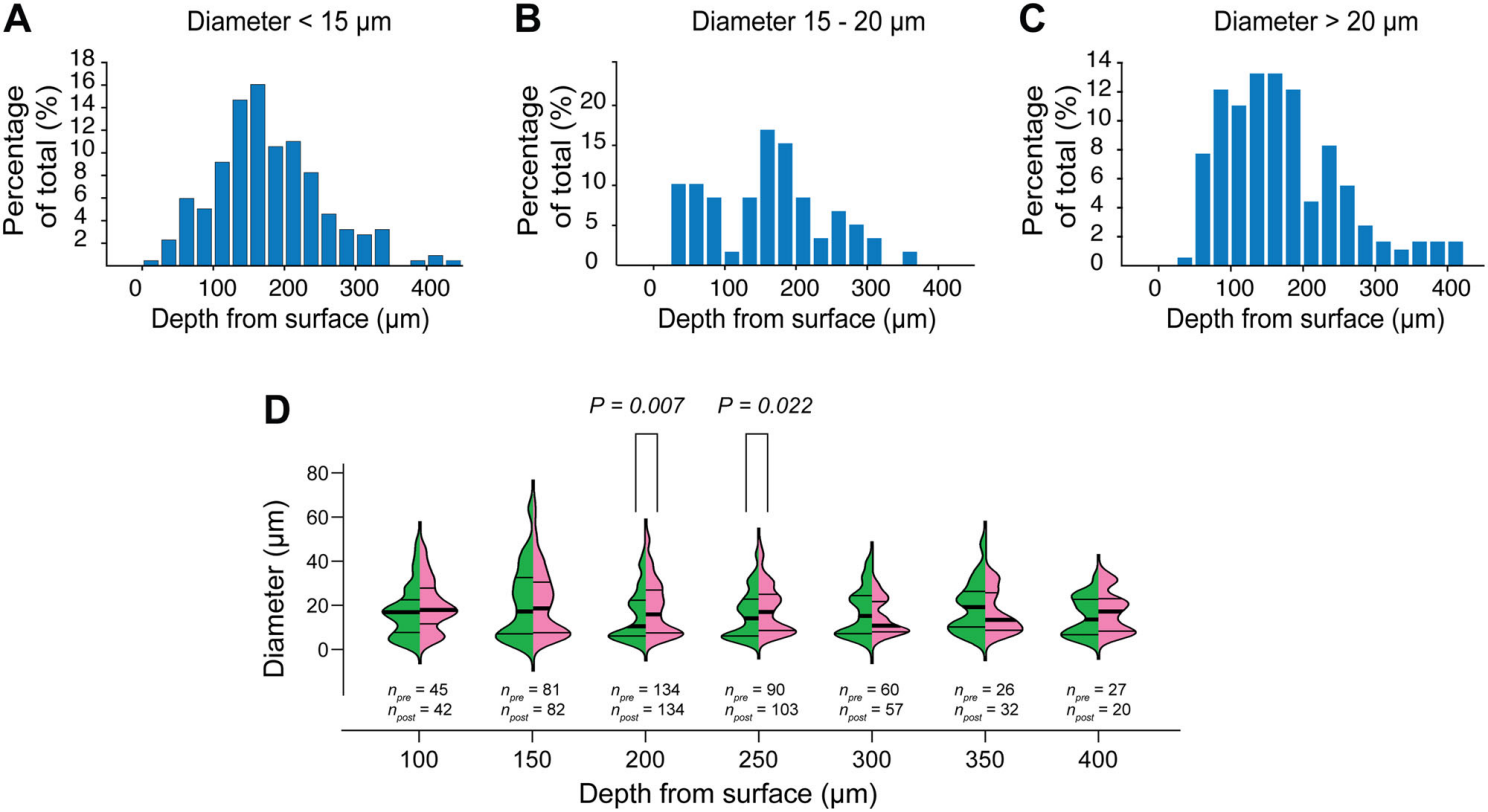
FUS-induced changes in vessel diameter, resolved in depth. **A**–**C** display depth-resolved histograms of vessel branches for the three ranges of branch diameter that were examined in Fig. 3C–E. **D** presents a depth-resolved analysis of FUS-induced vascular dilation. The violin plots show distributions of vessel branch diameters for branches positively identified as belonging to that specific depth range. This analysis extends 400 μm below the cortical surface, and individual bins span 50 μm. The data for all panels in this figure are derived from the results of 10 animals that were exposed to 10 W/cm^2^ FUS. Each violin plot is divided such that the green-shaded left half depicts the vessel branch diameter distribution prior to FUS, and the magenta-shaded right half depicts vessel branch diameter distributions post-FUS. The bold horizontal black bars within each violin plot represent the median and thin bars indicate lower and upper quartiles. Significance was measured using a Mann–Whitney–Wilcoxon test, and significant *P* values of the pre-post comparison are indicated above the brackets. The numbers of vessel branches that were included in pre- and post-FUS violin plots are indicated below the violins as *n*_pre_ and *n*_post_.

FUS-induced effects differed not only based on a vessel branch’s baseline diameter, but also based on depth. Figure 4A–C displays histograms of vessel depths for the three ranges of branch diameter that were examined in Fig. 3C–E. Because the distributions are markedly non-Gaussian, skewed, and possibly multimodal, the mean depths and quartiles for the three distributions overlap. However, it is visually apparent that vessel branches of diameter >20 μm are more weighted toward the surface compared with, at least, branches of diameter 15–20 μm. The skew in depth-dependence for branches of diameter >20 μm (Fig. 4C) within the span of 500 μm below the cortical surface is consistent with prior results from two-photon microscopy (e.g., Blinder et al.^62^).

This analysis also enabled us to identify depth-dependent changes in vessel branch diameter upon treatment with FUS. Figure 4D depicts depth-resolved branch diameter distributions for both pre and post sonication conditions. The results are binned in 50-μm increments, and for each bin, a split-violin plot shows the distribution before and after 10 W/cm^2^ (*I*_sppa_) sonication. Comparing individual depth bins, significant differences were found at depths of 200 and 250 μm (*P* = 0.007 and 0.022, respectively, using a Mann–Whitney–Wilcoxon test). The numbers of vessel branches that were included in pre- and post-FUS violin plots are indicated as well.

### Impact of FUS on vascular permeability and tissue integrity

**Fig. 5:**
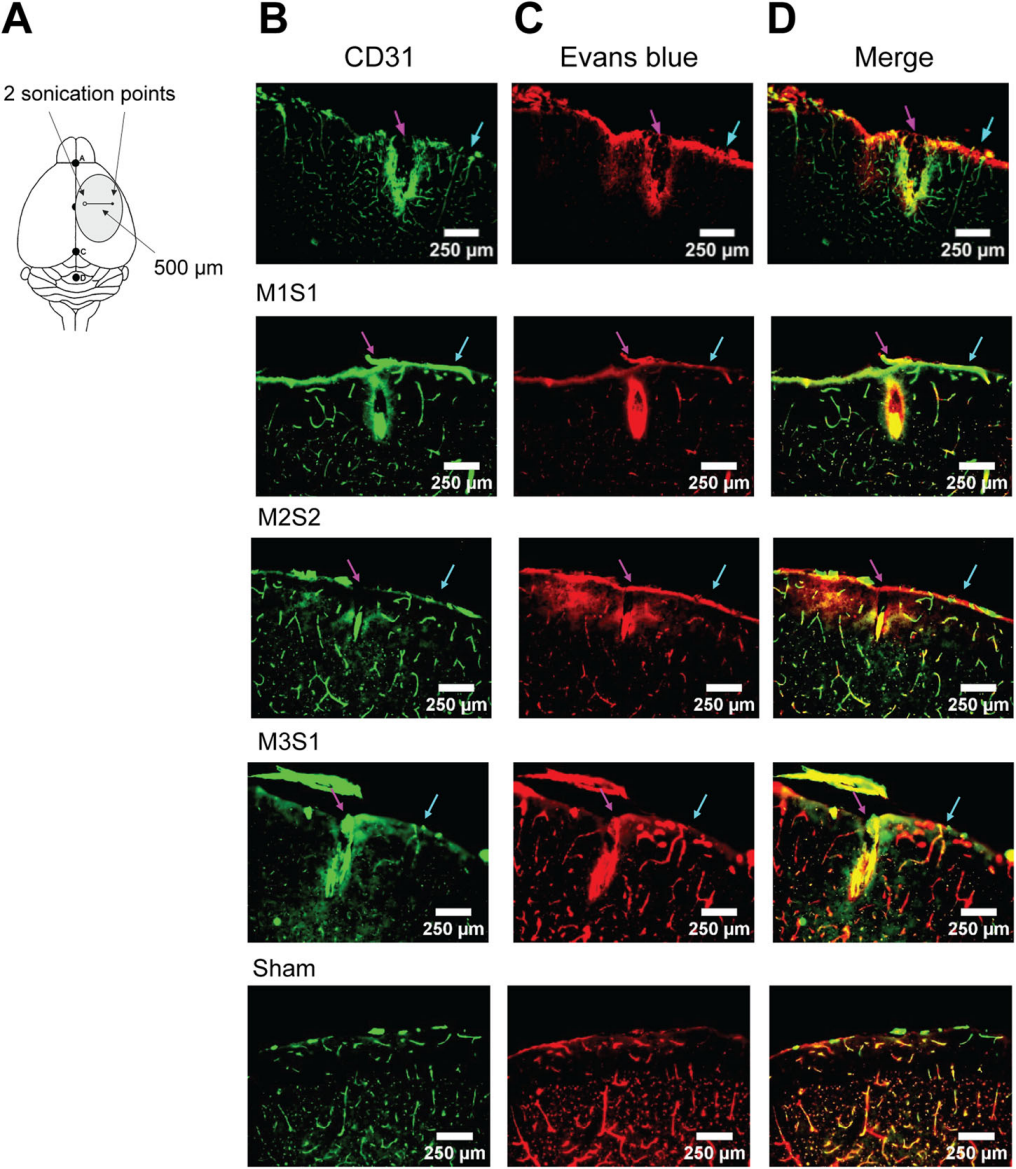
Immunohistochemical assessment of vascular integrity and BBB permeabilization. **A** shows an illustration of the relative coordinates on the brain where FUS was targeted. The medial location received higher intensity sonication (>200 W/cm^2^ *I*_sppa_, 100% duty cycle, 20 s), while the lateral location was sonicated at a lower intensity (1.2 W/cm^2^, 100% duty cycle for 20 s). **B**–**D** show coronal sections obtained at the rostrocaudal axis location where sonication was performed. The magenta and cyan arrows represent respectively, high- and low-intensity sonication locations. CD31 labeling is displayed in **B**, Evans blue in **C** and **D** shows a merged image. The results are presented for four sonicated animals (indicated by the experimental IDs that begin with “M”) and one sham animal.

At high intensities, FUS can permeabilize the blood-brain barrier (BBB) and impact neurovascular response^63^. To address the possibility that the vascular responses we observed were due to tissue damage or extravasation, in a separate cohort of animals (*N* = 5) we assessed vessel morphology and leakage of intravenously injected Evans blue dye, which binds to serum albumin and does not leave the vasculature unless the BBB is permeated. We exposed four of these mice (the remaining animal was used as an unsonicated sham) to FUS within the intensity range used in these experiments (1.2 W/cm^2^) and, as a positive control for tissue damage, high intensity (200 W/cm^2^), at adjacent locations on the same cortical hemisphere (Fig. 5). After 24 hours, sites exposed to high-intensity FUS displayed clear tissue damage as well as microvascular disruption, as visualized by vascular marker CD31. Regions targeted with high-intensity FUS also displayed extravasation of Evans blue dye in the parenchyma. These effects extended beyond 350 μm in depth. In contrast, exposure to a low intensity within the range of the other experiments (1.2 W/cm^2^) yielded no evidence of gross tissue disruption nor Evans Blue dye leakage.

## Discussion

The results we obtained fill a gap in the exploration of vascular responses to FUS, which have heretofore measured either response in larger vessels or in diffuse, bulk tissue hemodynamics. The closest relevant studies involved the use of two-photon microscopy to dissect microvascular changes following the combination of FUS and microbubbles^59,64,65^. Because microbubbles significantly amplify mechanical displacement due to volume oscillations, the underlying mechanisms are likely different in our study. The previous literature largely reports vasoconstriction, though Cho et al.^59^ also observed modest vasodilation in small-diameter vessel branches. The authors suggested that these effects could potentially reflect inflammatory responses due to BBB disruption^66,67^. Although our immunohistochemical analysis did not detect any significant Evans blue dye leakage indicative of BBB compromise, it remains possible that FUS induced a more subtle inflammatory effect.

The persistent microvascular dilation that we observed may well be a secondary response to FUS-induced or enhanced neuronal activity Clennell et al.^68^, for example, found that FUS can induce up to 8 hours of enhanced excitability in cultured cortical neurons, possibly due to dephosphorylation of select subunits of voltage-gated potassium channels (i.e., K_v_2.1). We have previously shown that acute, sensory activation evokes increases in capillary blood flow that subside within ~10 sec^69^. If the microvascular dilation we observed was secondary to neuronal effects, it, therefore, likely represents a prolonged vascular response tied to persistent neuronal excitability.

FUS-sensitive mechanosensitive ion channels provide another potential mechanism, as they are directly responsive to FUS in cultured cells and are expressed across neurovascular cell types^70^. While our experimental methods did not permit us to directly explore the involvement of specific cell types, the depth-resolved analysis of our imaging results may offer indirect supporting evidence for the role of pericytes. In the cortical microvasculature, the diameter of a vessel branch decreases as the “branch order” increases, which refers to the number of bifurcations from an individual penetrating arteriole that lead to that branch^71^. Branches of higher order feature sequentially different cell types; Grant et al.^72^, for example, observed that branch order in the mouse cortical vasculature predicts both the presence and type of pericytes found. Prior work that used two-photon imaging to identify vessel branching patterns found that arteriolar branching probability peaks at deeper layers (roughly depths beyond 200 μm) than venules, which peak within the first ~100 μm from the cortical surface^62^. In the context of this previous work, the diameter and depth biases that we observed suggest, albeit indirectly, that FUS-induced dilation occurs most prominently in high branch order pre-capillary arterioles and capillaries. It should be cautioned that conclusively resolving features in depth, such as branch order in OCT-A imaging is complicated by projection artifacts, or “tailing” underneath vessels which are caused by multiple scattering^73^. Consequently, while en face z-projections yield a clear portrait of the vasculature, individual depth ranges contain signals from depths above and below, significantly limiting the number of vessels that can be attributed to one particular depth range. Within narrow depth ranges, partial segments of vessel branches—particularly small diameter branches—are generally excluded as noise. This can leave small vessels under-represented in our depth-resolved analysis. These artifacts can be potentially mitigated using computational approaches such as convolutional neural network deep-learning techniques^74^. While this analysis is outside of the scope of the current report, future work will certainly pursue this line of investigation.

It should be noted that for the pooled results presented as violin plots in Fig. 4D, each animal had a different number of total vessels. While multivariate statistics are generally preferable for group comparisons, the limited number of vessel branches per animal—owing to the optical confounds described above—led us to use univariate approaches to enhance sensitivity in detecting significance. In the dataset depicted in Fig. 4, the number of depth-resolved vessels in each of 10 mice was within the same order of magnitude and varied from 23 to 89.

The prevalence of dilation in vessel branches likely containing pericytes may be especially relevant given the ongoing debate about their role in neurovascular coupling^75^. Recent findings suggest that pericytes are not only sufficient for maintaining basal capillary tone but may also facilitate dilation^76^. Importantly, pericytes demonstrate considerably slower response kinetics than smooth muscle cells, which is associated with their lower levels of α-smooth muscle actin (α-SMA) expression^77^. If the vessels most responsive to FUS are confirmed to lack smooth muscle cells, this would strongly suggest that altered pericyte function is a key mechanism. In this context, nitric oxide (NO) may play a key role given that FUS elicits NO release from astrocytes^31^ and likely activates endothelial NO synthase (eNOS)^78^. At the capillary level, an increase in NO concentration could cause dilation by relaxing pericytes^79^.

The use of anesthesia presents potential confounds for this and most other FUS neuromodulation studies in small animals. On their own, many anesthetics alter hemodynamics and vascular function through a wide range of mechanisms including altered cerebral autoregulation, vasomotor reactivity, and neurovascular coupling^80^. Yoo et al.^81^ found that FUS administered to the thalamus could alter anesthesia state, specifically for ketamine-xylazine. FUS administered to brainstem nuclei, particularly the dorsal raphe nucleus, has also been shown to accelerate recovery from isoflurane-induced anesthesia^82^. Decades of experimental and theoretical research have shown that systems-level thalamocortical oscillations and their interplay with brainstem nuclei serve a controlling role in regulating consciousness under anesthesia^83–91^. It is unsurprising, therefore, that directing FUS subcortically to those regions affects recovery from anesthesia. In our experiments, we targeted the somatosensory cortex to avoid subcortical effects, and isoflurane levels were kept below 2%, as used by He et al.^82^. Thus, while unlikely, some of our observations might reflect FUS influence on anesthesia state. Nevertheless, the lack of significant diameter changes in sham experiments suggests that post-FUS dilation did not result from global physiological trends over the experiment.

Ultimately, the broad concept of “persistent effects” owing to FUS neuromodulation likely reflects a conglomeration of neural, astrocytic, and vascular phenomena. At a basic level, the absence of dilation in larger vessels indicates that microvascular dilation does not simply reflect passive capillary recruitment. In the broader context of other studies, our findings are consistent with a pericyte-dependent mechanism, perhaps mediated by both mechanosensitive ion channel activation and NO release. It should be noted that while the current report focuses on morphological changes, future studies utilizing modified versions of our apparatus may be able to quantitatively report on blood flow changes. Hwang et al.^92^ for example, recently demonstrated the ability to extract surrogate indicators of blood flow in capillaries using an OCT device that employed a swept-source OCT system. Integrating flow-direction sensitive OCT-A could also help distinguish arterial vs. venous flow in the cerebrovascular networks. A better mechanistic understanding, including the development of computational models similar to those used for other non-invasive neuromodulation techniques (e.g., TMS, tDCS), would enhance experimental design and improve predictive accuracy for FUS-induced neuromodulation effects.

## Methods

### Surgical procedures

All animal experiments were performed in accordance with the guidelines of the Institutional Animal Care and Use Committee of New York Medical College, which also granted ethical approval for our study. We have complied with all relevant ethical regulations for animal use. Adult male C57BL/6 J mice 4–6 months of age were anesthetized with an initial dose of 5% isoflurane (vaporized in medical grade compressed oxygen) for <20 s and was later maintained at 1.5%. Following initial anesthesia induction, animals were positioned in a stereotaxic apparatus (Stoelting Co., Wood Dale, IL) and eyes were covered with petrolatum ophthalmic ointment (Puralube®, Fera Pharmaceuticals, Locust Valley, NY). Mice were administered buprenorphine (0.5 mg/kg, subcutaneously) and dexamethasone (0.2 mg/kg, subcutaneously) to reduce inflammation, and body temperature was measured and maintained at 37 °C with a closed-loop temperature-controlled heating system that included a heating pad (TC-1000, CWE, Ardmore, PA) and rectal probe (40-90-8D, FHC, Inc., Bowdoin, ME). Respiratory rate was monitored and maintained at ~100 breaths/min during the surgical and experimental procedures.

In preparation for the craniotomy procedure, the scalp was infused with lidocaine delivered subcutaneously, and a midline incision was performed on the region overlying the forelimb’s representation on the primary somatosensory cortex (at the same point on the rostrocaudal axis as bregma and ~2.5 mm lateral). A custom-fabricated aluminum headbar was affixed to the left side of the exposed skull with cyanoacrylate adhesive (Loctite 454, HenKel, Wisconsin) to improve positioning for surgical and experimental procedures. A 2.5 × 3 mm rectangular craniotomy was made using a handheld dental drill (Osada Model EXL-M40), being careful not to damage the dura mater. To maximize combined optical and acoustic transparency, the exposed brain was covered with a 2.5 × 2.5 mm square piece of poly(4-methyl-1-pentene) (PMP) (Goodfellow Cambridge Ltd, Huntingdon, England) (Koekkoek et al., 2018). The gap between the outer edge of the PMP window and the inner edge of the craniotomy was filled with colorless surgical silicone adhesive (Kwik-Sil, World Precision Instruments, Florida), and the PMP window was sealed to the skull with instant adhesive gel (Loctite 454, HenKel, Wisconsin). The metal headbar and exposed skull were covered with dental cement (C&B Metabond, Parkell, New York). Animals were allowed to recover for four days before imaging and sonication experiments and euthanized directly following imaging sessions via cervical dislocation.

### OCT-A system and data acquisition

We built a customized Spectral Domain OCT shown in Fig. 1A as our OCT-A device. The system incorporates a superluminescent diode light source (Superlum, cBLMD‐T‐850‐HP, ${\lambda }_{c}=850{nm}$, $\Delta \lambda =160{nm}$) and a spectrometer (Cobra-S 800, Wasatch Photonics) with a line-scan camera (Teledyne e2v) operating at 80 kHz. A 5X objective lens (M Plan Apo NIR, Mitutoyo) with a long working distance was installed in the sample arm. The system’s axial and lateral resolution are ~2.5 µm and ~3.5 µm in tissue, respectively. As depicted in Fig. 1A, there was an air gap between the objective lens and the entry window of the ring transducer; we compensated for this by adjusting the path length of the reference arm of the OCT system.

Custom OCT system software written in C++ and CUDA was developed to control hardware, including laser control, galvanometers for scanning, and cameras for image acquisition. The OCT images were acquired and processed in near real-time with GPU processing on a high-performance graphic card (GeForce RTX 3090, NVIDIA). A CUDA-based accelerated DFT registration and Split Spectrum Amplitude Decorrelation algorithm^93^ was developed to produce OCT-A images. Angiography processing separates the static reflectance signal from the higher dynamic OCT signal that arises from flowing blood, resulting in relative blood flow images. Each 3D OCT anatomical volumetric snapshot contained 600 × 600 cross-sectional planes (A-scans × B-scans). repeated frames per B-scans over the same location were acquired for OCT-A. The total acquisition and GPU processing time for each angiographic volume was 30 s. En face projections were generated from flattened OCT-A images using MATLAB (MathWorks, Natick, MA).

### In vivo sonication protocol

A total of 28 animals were used in experiments including 10 animals sonicated with 1 W/cm^2^, 10 animals sonicated with 10 W/cm^2^, 3 sham animals, and 5 animals used separately for immunohistochemical analysis only. Each animal was used in only one recording session. Animals were induced with 5% isoflurane, then maintained at 1–1.5%. Respiration was monitored continuously and anesthesia depth was assessed periodically via toe-pinch pedal reflex. As depicted in Fig. 3D, in each recording session, mice were sonicated twice with the same intensity to explore not only a single sonication effect, but to capture potentially cumulative effects of multiple doses. To minimize overlap of acute responses, repeated sonication doses were delivered with a 10-minute separation. This timing was informed by prior reports that acute hemodynamic responses largely subside within 10 seconds of sonication^56,57,94^. Experimental results from these experiments were included in analysis if the animal survived and maintained a stable plane of anesthesia and physiological state during full course of the recording session, which typically lasted 40 minutes from induction to completion. Sham experiments were performed on separate cohorts of animals that were subject to surgical procedures (including implantation, recovery periods, and anesthesia protocol) that were identical to animals used in experiments that involved sonication. This both OCT-A imaging experiments as well as the separate cohort used for immunohistochemical assessment. For functional sonication experiments, animals were situated under the ultrasound transducer prepared the same as it was for sonication experiments (i.e., filled with de-gassed water and coupled to the head with ultrasound gel); however, sonication was not applied. OCT-A imaging was performed at the same exact time periods as those in experiments with sonicated animals. Statistical analysis was performed using GraphPad Prism-10 (GraphPad Software, CA, USA). Specific tests used for different analyses are described explicitly in the text.

### Combined optical and acoustic apparatus

To permit simultaneous FUS application and OCT-A angiographic portraits of blood flow, we used a custom mount that permitted fine, 3-axis positioning for a wide-aperture FUS ring transducer (customized H-205B, Sonic Concepts, Inc., Bothell, WA), described in ref. ^95^. The FUS transducer’s fundamental frequency was 510 kHz and achieved a focal spot of lateral width ~3.3 mm (full-width at half-maximum), which effectively overfilled the field of view. For the OCT-A modality, the FUS transducer was integrated into the beam path ~2 cm below the primary objective lens. Animals were positioned on a platform atop a lab jack for coarse adjustments (Thorlabs, Inc., NJ). Stimulus waveforms were generated and amplified by a TPO-106 transducer drive system (Sonic Concepts, Inc., Bothell, WA). Sonication was delivered as a 20 s burst 510 kHz ultrasound delivered as a series of 0.5 ms pulses at a repetition rate of 1 kHz (i.e., 50% duty cycle). Two intensities were used, a low of 1 W/cm^2^ and a high of 10 W/cm^2^ (both *I*_sppa_). This yielded peak pressures of 0.124 MPa and 0.45 MPa, respectively. Beam properties were characterized in a tank of de-gassed water using a RESON spherically directional hydrophone.

To confirm the co-localized acoustic and optical foci, we used a thermochromic liquid-crystalline sheet (peak thermochromic range 30–35 °C) to visualize the FUS focus centroid. Because the OCT illumination center wavelength was near-infrared, the scanned beam OCT beam could be resolved as a “crosshairs” on the thermochromic sheet to correct for any mismatch in position relative to the FUS focus.

### Image processing and analysis methods

Image stacks were first processed to remove background noise and co-register to account for any positioning drift between repeated scans. We utilized Top-hat filtering (6.7-μm window width) to segment blood vessels and bin by diameter, then applied functions from the Vessel Analysis plugin in ImageJ (Fiji)^96^ for arterioles (15–20 μm diameter) for binarization and skeletonization. Smaller vessel analysis utilized additional functions from the VasoMetrics toolbox, which has been verified for use in quantifying both OCT-A and two-photon microscopy data^97^. A visualization of the vessel segmentation analysis is depicted in Supplementary fig. 1, which also illustrates that vessel branches could be tagged and tracked individually throughout the course of FUS application. Given the OCT-A system’s resolution limitations, the minimum inclusion diameter of 5 μm. To mitigate the inherently lower signal-to-noise ratio for vessel branches of small diameter, the average diameter was calculated based on the average of a series of finely spaced, automatically selected cross-sectional lines. Cross-sectional line spacing along vessel branches was typically 5 μm, or ~15 lines for the smallest vessel branches.

To best resolve the depth profile of vessels (Fig. 4), we axially binned image volumes into 25-μm divisions in-depth and performed vessel segmentation within those narrow ranges. Because the surface of the brain is neither perfectly flat nor precisely perpendicular to the scanning beam, prior to this processing, image volumes were de-tilted using custom Matlab code^98^. This helped ensure that our depth characterization of vasculature was as accurate as possible.

### Immunohistological procedures

Brains were dissected out and post-fixed in 4% paraformaldehyde for 24 h at 4 °C, after which they were washed in phosphate-buffered saline solution (PBS) for 1 hour then equilibrated in 30% sucrose. Brains were then embedded in OCT compound (Tissue-Tek, Sakura Finetechnical Co., Tokyo, Japan) and sectioned into 18 µm thick coronal sections on a cryotome. Sections were washed and blocked by incubation with 10% normal goat serum (NGS) in PBS supplemented with 0.4% Triton X-100 for 1 h at room temperature. They were then incubated with CD31 Rat Monoclonal Antibody (PECAM-1) (1:500 dilution, Abcam 56299) in 2% NGS and 0.3% Triton X-100 at 4 °C overnight. Sections were then incubated for an hour with Alexa-488 labeled secondary antibody (1:500 dilution, goat anti-rat, Abcam150165), after which sections were mounted on slides, dehydrated, and coverslipped with non-fluorescent mounting medium (Krystalon, 64969-95, EMD, Darmstadt, Germany).

BBB disruption was visualized based on the leakage of intraperitoneally administered Evans blue dye into the brain’s parenchyma. Half an hour prior to FUS treatment, 0.1 mL of a 1% solution of Evans blue dye was injected intraperitoneally. FUS was administered as described above for assessing changes in CD31 distribution. The brain was then dissected out and post-fixed, then prepared as described above for CD31 analysis.

Distribution of Evans blue dye and CD31 was observed using fluorescence imaging with an All-in-One Fluorescence Microscope (Keyence, BZ-X810, Osaka, Japan). The illumination and emission filter parameters for observing Evans blue were 560 ± 28 nm (excitation) and 645 ± 38 nm (emission); for CD31, filter parameters were 470 ± 40 nm excitation, 525 ± 50 nm emission).

### Statistics and reproducibility

The significance of the observed effects of sonication, depicted in Fig. 3, was assessed using two-way ANOVA and Fisher’s Least Significant Difference test. For depth-resolved analysis, significance was assessed using a Mann–Whitney–Wilcoxon test. Overall, this study presents the experimental results of 28 animals, which include 10 mice per cohort for each sonication intensity (1 W/cm^2^ and 10 W/cm^2^), 3 sham experiments, and 5 animals used separately and exclusively for immunohistochemical analysis of sonication effects.

### Reporting summary

Further information on research design is available in the Nature Portfolio Reporting Summary linked to this article.

## Supplementary information

Supplementary Information

Description of Additional Supplementary Files

Supplementary Data 1

Supplementary Data 2

Supplementary Data 3

Reporting summary

## Data Availability

The datasets obtained and analyzed during the current study accompany this article as Supplementary Data files.

## Code availability

The custom code used for analysis in this study can be freely accessed at 10.5281/zenodo.14249219.
