# Supplementary material for: Label free, capillary-scale blood flow mapping in vivo reveals that low-intensity focused ultrasound evokes persistent dilation in cortical microvasculature
Source: Commun Biol. 2025 Jan 6;8:12. doi: 10.1038/s42003-024-07356-2 (PMC11704147; doi:10.1038/s42003-024-07356-2)
Supplement: Supplementary file 1 — Supplementary Information [file 42003_2024_7356_MOESM1_ESM.pdf]

Supplementary Information

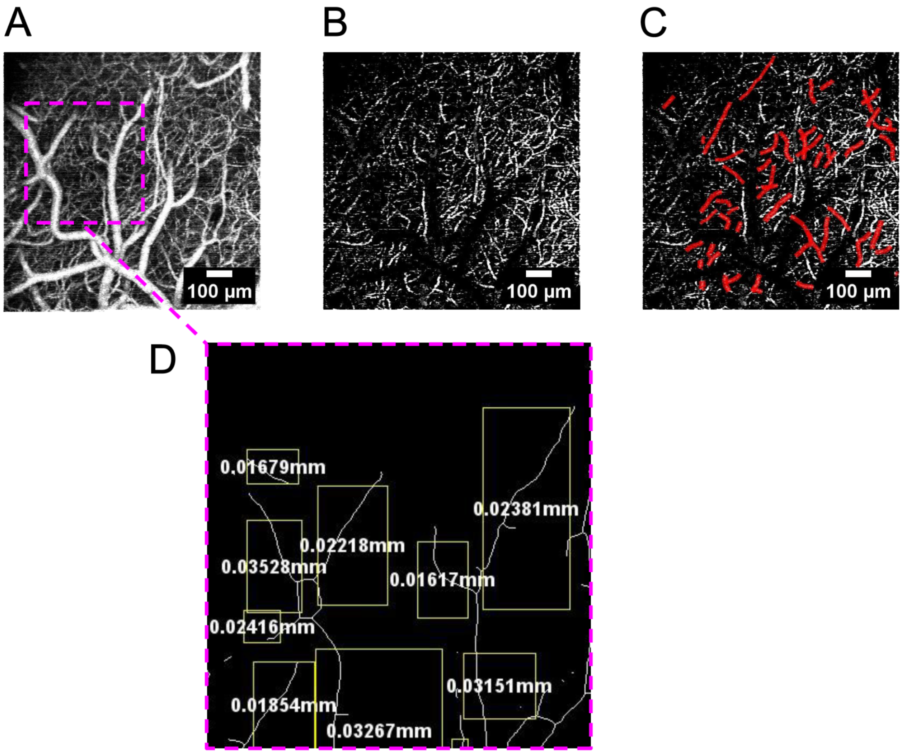

Supplementary Figure 1: Diameter-specific analysis of angiographic measurements. **A** depicts a *z*-projection of a 1 mm × 1 mm × 540 µm region of somatosensory cortex *in vivo*. **B** illustrates diameter-specific segmentation of microvascular diameter vessel branches; red vessel branches in **C** were auto-selected for analysis because they could be resolved throughout all conditions. **D** illustrates, in a magnified subregion of **A**, the vessel "tagging" feature of our data analysis pipeline, which enabled us to track individual vessel branches through the course of FUS application.

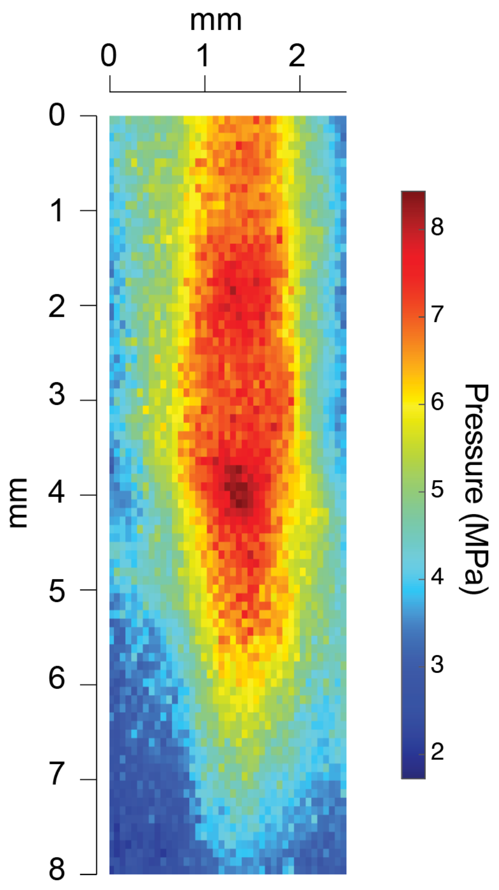

Supplementary Figure 2: Axial pressure profile of the FUS beam, characterized in a tank of degassed water using a RESON spherically directional hydrophone. The horizontal axis corresponds to the plane parallel to the surface of the imaged surface, and the vertical axis corresponds to axial depth in the tissue. On the vertical scale, “0” corresponds to the bottom exit plane of the ring transducer, which is above the mouse’s head because the transducer’s focal curvature begins within the ring. The mouse’s skull was positioned at the beam’s actual focus.
