# Supplementary material for: Label free, capillary-scale blood flow mapping in vivo reveals that low-intensity focused ultrasound evokes persistent dilation in cortical microvasculature
Source: Commun Biol. 2025 Jan 6;8:12. doi: 10.1038/s42003-024-07356-2 (PMC11704147; doi:10.1038/s42003-024-07356-2)
Supplement: Supplementary file 2 — Description of Additional Supplementary Files [file 42003_2024_7356_MOESM2_ESM.pdf]

Description of Additional Supplementary Files

File name: Supplementary Data 1.xlsx

Description: The source data for Figure 2D.

File name: Supplementary Data 2.xlsx

Description: The source data for Figure 3B, C, D, & E.

File name: Supplementary Data 3.xlsx

Description: The source data for Figure 4.
